# Spatial Metabolomics Profiling Reveals Curcumin Induces Metabolic Reprogramming in Three-Dimensional Tumor Spheroids

**DOI:** 10.3390/metabo14090482

**Published:** 2024-09-02

**Authors:** Zihan Zhu, Yaqi Zhang, Lei Wang, Haoyuan Geng, Min Li, Shiping Chen, Xiao Wang, Panpan Chen, Chenglong Sun, Chao Zhang

**Affiliations:** 1 Key Laboratory for Natural Active Pharmaceutical Constituents Research in Universities of Shandong Province, School of Pharmaceutical Sciences, Qilu University of Technology (Shandong Academy of Sciences), Jinan 250014, China; 2 Key Laboratory for Applied Technology of Sophisticated Analytical Instruments of Shandong Province, Shandong Analysis and Test Center, Qilu University of Technology (Shandong Academy of Sciences), Jinan 250014, China; 3 School of Pharmacy, Shandong University of Traditional Chinese Medicine, Jinan 250355, China; 4 Department of Pediatrics, Qilu Hospital of Shandong University, Jinan 250012, China

**Keywords:** spatial metabolomics, metabolic reprogramming, curcumin, tumor spheroids

## Abstract

Curcumin is widely recognized for its diverse antitumor properties, ranging from breast cancer to many other types of cancers. However, its role in the tumor microenvironment remains to be elucidated. In this study, we established a 3D tumor spheroids model that can simulate the growth environment of tumor cells and visualized the antitumor metabolic alteration caused by curcumin using mass spectrometry imaging technology. Our results showed that curcumin not only exerts a profound impact on the growth and proliferation of breast cancer cells but in situ multivariate statistical analysis also reveals the significant effect on the overall metabolic profile of tumor spheroids. Meanwhile, our visualization map characterized curcumin metabolic processes of reduction and glucuronidation in tumor spheroids. More importantly, abnormal metabolic pathways related to lipid metabolism and polyamine metabolism were also remodeled at the metabolite and gene levels after curcumin intervention. These insights deepen our comprehension of the regulatory mechanism of curcumin on the tumor metabolic network, furnishing powerful references for antitumor treatment.

## 1. Introduction

Breast cancer (BC) dominates the incidence of cancer in women [1], stemming from malignant cells in breast tissue and distinguishing phenotypes such as invasiveness, metastasis, and migration [2,3]. However, its drug resistance poses challenges for conventional treatment protocol by means of chemotherapy and targeted therapies [4]. Notably, metabolic reprogramming has been increasingly linked to drug resistance in cancer therapy. BC cells manipulate signaling pathways and metabolic processes to drive phenotypes to sustain proliferative survival demands [5,6,7]. Metabolic reprogramming may provide novel biomarker clues for cancer therapeutic interventions [8]. Growing evidence shows that targeting fatty acid synthase and glutaminase exhibits antitumor activity, inspired by the upregulation of fatty acid de novo synthesis and glutamate synthesis via oncogenic signals [9,10]. Therefore, an in-depth investigation of metabolic reprogramming is of great significance to elucidate the molecular underpinnings of breast cancer initiation and progression, offering valuable implications for the discovery of novel therapeutic targets.

Curcumin, a polyphenolic compound isolated from the rhizome of *Curcuma longa* L., possesses a variety of biological activities, including antibacterial, anti-inflammatory, proapoptotic, and antioxidant properties. Notably, curcumin demonstrates considerable potential in treating various cancers, particularly in the treatment of breast cancer [11,12]. Its effectiveness can be attributed to its ability to modulate key tumor pathways, such as EGFR, NF-κB, and HIF1α [13,14], which are critical in the progression and survival of tumors, highlighting the significant role of curcumin in cancer intervention. However, the investigation of the impact of curcumin on breast cancer has predominantly been conducted in two-dimensional cell culture systems, which do not fully mimic the complex three-dimensional architecture and microenvironment of in vivo tumors. Three-dimensional (3D) multicellular tumor spheroids provide a more realistic model, which exhibit oxygen and nutrient gradients, better simulate the tumor microenvironment [15,16], and yield more accurate responses to drugs. This potentially leads to more effective and relevant insights in breast cancer research, especially regarding the impact of curcumin.

Metabolomics facilitates a comprehensive approach to studying drug mechanisms by analyzing the global pattern of change in metabolic network end products. It brings a new perspective to the evaluation of drugs through exploring drug metabolism processes, such as identifying drug targets, detecting drug action patterns, predicting drug responses, and exploring drug interactions [17,18,19,20]. However, conventional assays, such as LC-MS and GC-MS, disregard spatial information of metabolites due to tissue homogenization, molecular extraction, separation, and purification during the preprocessing stage in heterogeneous samples [21,22,23]. In contrast, mass spectrometry imaging (MSI)-based metabolomics has enabled in situ analysis of metabolites [24,25]. For instance, spatial metabolomics has reflected crucial information about metabolic characteristics and regulatory networks of diseases within cell spheroids, mice, zebrafish, cancer tissues, etc., providing a more direct and accurate understanding of the multifaceted mechanisms underlying disease onset and progression [26,27,28,29,30]. The application of spatially resolved metabolomics combined with 3D tumor spheroids provides a robust technological platform for exploring the mechanisms of curcumin from the metabolic perspective. This approach enables a deeper understanding of how curcumin influences metabolic pathways within the complex microenvironment of tumor spheroids, offering valuable insights into its therapeutic potential and efficacy.

In this study, we investigated the antitumor effects of curcumin on MDA-MB-231 cells, specifically examining their impact on life processes such as proliferation, migration, invasion, and apoptosis. To better comprehend the metabolic alterations induced by curcumin, we employed spatially resolved metabolomics technology to visually depict the metabolic remodeling within 3D-cultured cancer spheroids, which simulate in vivo tumor characteristics. Our findings highlight the significant influence of curcumin on lipid metabolism, including fatty acids and phosphatidic acids, as well as polyamine synthesis and metabolism, as represented by glutamine and glutamate. This metabolic reprogramming has profound effects on the energy metabolism and life course of cancer cells. Our study may not be comprehensive, but we seek to briefly evaluate the antitumor effects of curcumin, with a view to screening for potential metabolic markers and effective natural products to enhance antitumor treatment.

## 2. Materials and Methods

### 2.1. Chemicals and Reagents

1,5-DAN, curcumin, and tetrahydrocurcumin were purchased from Shanghai Aladdin Bio chemical Technology Co., Ltd. (Shanghai, China). Curcumin and tetrahydrocurcumin were dissolved in dimethyl sulfoxide (Sigma, St. Louis, MO, USA) for use. The human MDA-MB-231 breast cancer cells were sourced from the National Infrastructure of Cell Line Resource (Shanghai, China). The MTT Assay Kit was obtained from Shanghai Beyotime Biotechnology Co., Ltd. (Shanghai, China).

### 2.2. Cell Culture

Human MDA-MB-231 breast cancer cells were cultured in high-glucose DMEM medium (Gibco, Grand Island, NY, USA), supplemented with 10% FBS, 100 IU/mL penicillin, and 100 μg/mL streptomycin. The cells were cultured in a 37 °C constant temperature incubator containing 5% carbon dioxide for three generations of stable cell transmission. Cells in the logarithmic growth phase were chosen for subsequent experiments.

### 2.3. Cell Viability

Two-dimensional cell viability was determined by the MTT Assay Kit. Cells were seeded in a 96-well plate at a concentration of 2 × 10^5^ cells/mL with 100 μL per well. The drug group and blank group were set up, respectively. The concentrations of curcumin in the drug groups were 12.5 μM, 25 μM, 50 μM, and 100 μM, while the blank group received an equal volume of DMSO. Each group was prepared with 4 replicates. After 24 h of cell adhesion, the cells were treated with curcumin or DMSO separately. After 72 h of incubation, measurements were performed in accordance with the instructions. 

Three-dimensional tumor spheroids viability was determined by the CellTiter Glo^®^3D Cell Viability Assay (Promega Corporation, Madison, WI). Cells in the logarithmic growth stage were digested, and 10% Matrigel (Corning Inc., Bedford, MA, USA) was used to make a cell suspension with a concentration of 2 × 10^5^ cells/mL. Cell suspension was added to V-bottom 96-well plates (Sumitomo Bakelite, Tokyo, Japan) 100 μL per well (*n* = 4). This was centrifuged at 1500 rpm for 10 min and incubated in an incubator for 48 h. After 72 h of drug treatment, the activity of the 3D tumor spheroids’ viability was measured according to the instructions.

### 2.4. Cell Migration

Cell suspensions at a concentration of 1.5 × 10^5^ cells/mL were inoculated in labeled 6-well plates at 2 mL per well. After 24 h of cell growth, a 10 μL sterilized pipette tip was used to perform a scratch operation perpendicular to the plate. We discarded the original medium and washed with PBS, and then each group was treated with complete medium containing different concentrations of curcumin and DMSO. Photographs were taken at 0 h and 24 h, respectively, for statistical analysis. The area of the blank section is denoted as “S”. The migration area calculation formula is as follows: Migration area (%) = 1 − S_24h_/S_0h_.

### 2.5. Cell Invasion

The invasive ability of MDA-MB-231 cells was assessed using 8 μm pore size transwell chambers (Costar, Cambridge, MA, USA). The chambers were pre-coated with a diluted Matrigel and incubated at 37 °C for 4 h to form a gel, hydrated with serum-free medium for 30 min, and then prepared for use. A cell suspension at a concentration of 4 × 10^5^ cells/mL was added to the upper chamber, while the lower chamber received 750 μL of complete medium with DMSO or varying concentrations of curcumin. Twenty-four hours later, the cells in the upper chamber were fixed with 4% paraformaldehyde. We stained the cells with 0.1% crystal violet and removed the upper layer of the cells. The cells that had invaded through the lower chamber were counted and photographed.

### 2.6. Caspase 3 Activity 

Caspase 3 activity was determined by using the Caspase 3 Activity Assay Kit (Beyotime, Nanjing, China). Lysate was used to lyse the cells at a ratio of 2 × 10^4^ cells/μL. After lysis was completed, we performed centrifugation and collected the supernatant according to the manufacturer’s protocol. The protein content of each group was also determined using the Quick Start™ Bradford Protein Assay (Bio-Rad Laboratories, Inc., Hercules, CA, USA).

### 2.7. MALDI-MSI Analysis

Three-dimensional tumor spheroids were washed with PBS, embedded with OCT, and cut into sections of 12 μm thickness in a cryostat microtome (Thermo CryoStar NX50 NOVPD, Bremen, Germany). A DAN solution with a concentration of 2.5 mg/mL was prepared using ACN/H_2_O (8:2) as the matrix solution. Sections were fixed on the HTX TM-Sprayer™ (HTX Technologies, Carrboro, NC, USA) and finely sprayed with DAN solution for 10 cycles. The flow rate was set to 0.075 mL/min, and the nozzle movement speed was set to 800 mm/min. Then, the sample (*n* = 3) was detected in a rapifleX^TM^ MALDI TOF/TOF mass spectrometer (Bruker Daltonics, Billerica, MA, USA). Sample detection was performed in positive- and negative-ion mode, respectively. The spatial resolution was 20 μm, laser energy was 65%, laser frequency was 5000 Hz, laser accumulation was 200 times, and mass spectrometry data were obtained at *m/z* 70~1000.

### 2.8. Analyte Identification

The extracted adducted ions were first compared with the Metlin database (http://metlin.scripps.edu, accessed on 22 September 2023.) with a mass error of <10 ppm. Then, the ions of interest were extracted to perform high-resolution LC-MS/MS analysis using the Bruker Impact II-ESI-Q-TOF mass spectrometer (Bruker Daltonics, Billerica, MA, USA) and on-tissue MS/MS using the rapifleX^TM^ MALDI TOF/TOF mass spectrometer (Bruker Daltonics, Billerica, MA, USA). Structural identification was carried out according to high-resolution tandem MS data and standard MS/MS spectra in the Metlin database.

### 2.9. Quantitative Real-Time PCR (qRT-PCR) Assay

The Spin Column Animal Total RNA Purification Kit (Sangon Biotech, Shanghai, China) was used for cell purification and RNA extraction. The cDNA was synthesized using the AMeasy 1st Strand cDNA synthesis kit (ALLMEEK, Beijing, China), and q-PCR was performed by using the 2 × PerfectHS SYBR QPCR Mixture kit (ALLMEEK, Beijing, China). The relative quantification of the mRNA levels of each gene between groups was calculated by the 2^−ΔΔCt^ method and normalized using the β-actin gene as a control. Primer information is shown in Supplementary Table S1.

### 2.10. Data Processing

GraghPad Prism 9 software and Image J2 software were used for data statistics and analysis, and the data for each group were expressed as mean ± standard deviation (SD). One-way ANOVA and Student’s t-test were used for comparative analysis of the groups, with the significance threshold set at *p* < 0.05. MALDI-MSI images were constructed using SCiLS Lab 2018b software (GmbH, Bremen, Germany). The MALDI-MS spectra of regions of interest (ROIs) were extracted to perform in situ principal component analysis (PCA) and probabilistic latent semantic analysis (PLSA) in SCiLS Lab 2018b software using the component analysis tool. All images were normalized to total ion counts, and each of the PCA and PLSA spots represent a mass spectrometry imaging pixel.

## 3. Results and Discussion

### 3.1. Curcumin Inhibits Cell Viability of MDA-MB-231 Cells

**Figure 1:**
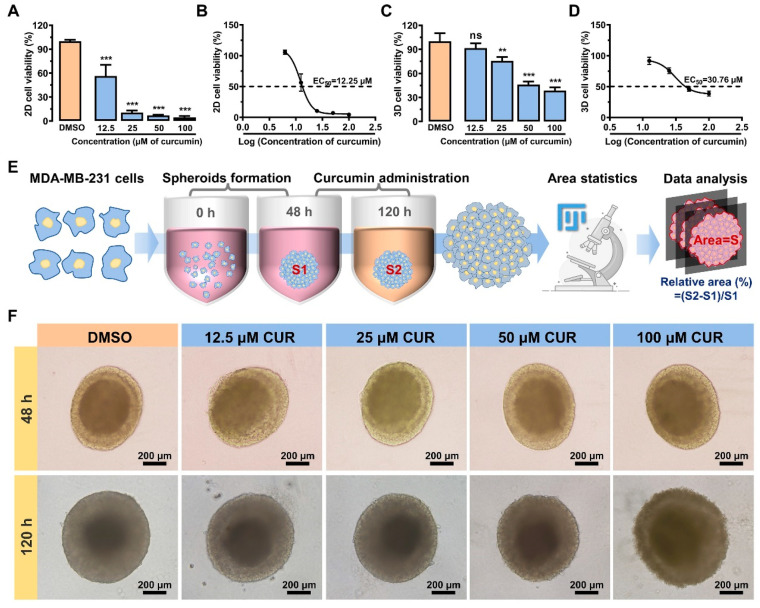
The inhibitory effect of curcumin in 2D-cultured and 3D-cultured MDA-MB-231 cells. (**A**) Cell viability of 2D MDA-MB-231 cells after intervention with curcumin. (**B**) EC_50_ of 2D MDA-MB-231 cells treated with curcumin. (**C**) Cell viability of 3D MDA-MB-231 tumor spheroids after intervention with curcumin. (**D**) EC_50_ of 3D MDA-MB-231 tumor spheroids treated with curcumin. (**E**) Three-dimensional tumor spheroids cultivation and data processing diagram. (**F**) The growth of spheroids after intervention with different concentrations of curcumin. Data are expressed as mean ± SD (*n* = 4). ** *p* < 0.01, *** *p* < 0.001, ns: not significantly different.

Cell viability serves as a valuable indicator for assessing the impact of drugs on the physiological state of cells. In order to investigate the effects of curcumin and tetrahydrocurcumin on triple-negative breast cancer, we performed the MTT assay to determine the viability of the MDA-MB-231 cells in monolayer culture. Figure 1A,B demonstrated that curcumin significantly suppressed cell viability at concentrations of 12.5 μM, 25 μM, 50 μM, and 100 μΜ, with an EC_50_ value of 12.25 μM. The antitumor cancer activity of curcumin increased proportionally with its concentration, showing a dose-dependent manner. In contrast, tetrahydrocurcumin only evinced inhibition at higher doses (Figure S1A), suggesting a more pronounced inhibition of curcumin on tumor cells. Subsequently, we utilized 3D-cultured spheroids to assess the impact of curcumin on cell viability. The results (Figure 1C) showed that even at a concentration as low as 25 μM, curcumin exhibited potent inhibitory properties on spheroid viability compared to the DMSO group. Furthermore, the EC_50_ of curcumin on 3D tumor spheroids is 30.76 μM (Figure 1D), notably higher than that of 12.25 μM in the 2D culture. The culture process and growth status of the tumor spheroids are shown in Figure 1E,F. Despite potential differences in spheroid morphology, the relative increase in spheroid area (Figure S1B) provided convincing evidence for the ability of curcumin to curb spheroid growth with an increasing concentration. Overall, our results showed that curcumin effectively inhibits the cell viability of MDA-MB-231 cells, while 3D tumor spheroids exhibit a higher tolerance to curcumin compared to 2D cell cultures.

### 3.2. Curcumin Inhibits the Growth and Metastasis of MDA-MB-231 Cells

**Figure 2:**
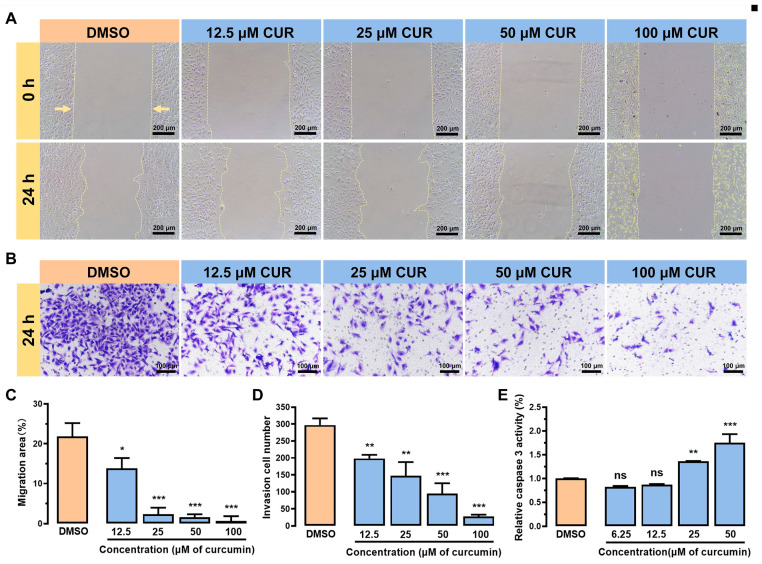
Curcumin affects the migration, invasion, and apoptosis of MDA-MB-231 cells. (**A**) The effect of curcumin on the migration of MDA-MB-231 cells. The yellow arrows indicate the approximate migration direction of cells. (**B**) The effect of curcumin on the invasion of MDA-MB-231 cells. (**C**) The migration area of MDA-MB-231 cells. (**D**) Quantitative analysis of MDA-MB-231 cell invasion. (**E**) The effect of curcumin on caspase 3 activity in MDA-MB-231 cells. Data are expressed as mean ± SD (*n* = 4). * *p* < 0.05, ** *p* < 0.01, *** *p* < 0.001, ns: not significantly different.

Cell migration and invasion are crucial processes in tumor metastasis, as they are of great significance in elucidating how tumor cells escape from the primary tumor and establish metastatic lesions in distant locations. To evaluate the impact of curcumin on the migration of MDA-MB-231 cells, we validated it through scratch experiments. Cell scratch experiments obtain migration rates by creating scratches and comparing images of where cell migration begins and ends [31]. Compared with the DMSO group, 12.5 μM of curcumin could significantly affect the migration of MDA-MB-231 cells (Figure 2A,C). Additionally, transwell was used to simulate the process by which tumor cells infiltrate the surrounding tissue by breaking through normal interstitial barriers, and fetal bovine serum (FBS) was added to the lower chamber as a chemokine to induce the invasion of MDA-MB-231 cells. In agreement with Figure 2B, the invasiveness of MDA-MB-231 cells was significantly reduced (*p* < 0.001) at curcumin concentrations of 12.5 μM, 25 μM, and 50 μM (Figure 2D) in a concentration-dependent manner. Caspase 3 is a key enzyme in the apoptosis process and plays an important role in the regulation of cell survival and death. Figure 2E shows that caspase 3 activity was induced upon curcumin treatment, with 25 μM curcumin significantly promoting caspase 3 expression and inducing apoptosis in MDA-MB-231 cells compared to the DMSO group. Taken together, the series results showed that curcumin can inhibit the growth and metastasis of tumor cells via modulating the migration, invasion, and apoptosis of MDA-MB-231 cells.

### 3.3. Spatial Distribution of Curcumin and Its Metabolites in 3D Tumor Spheroids

**Figure 3:**
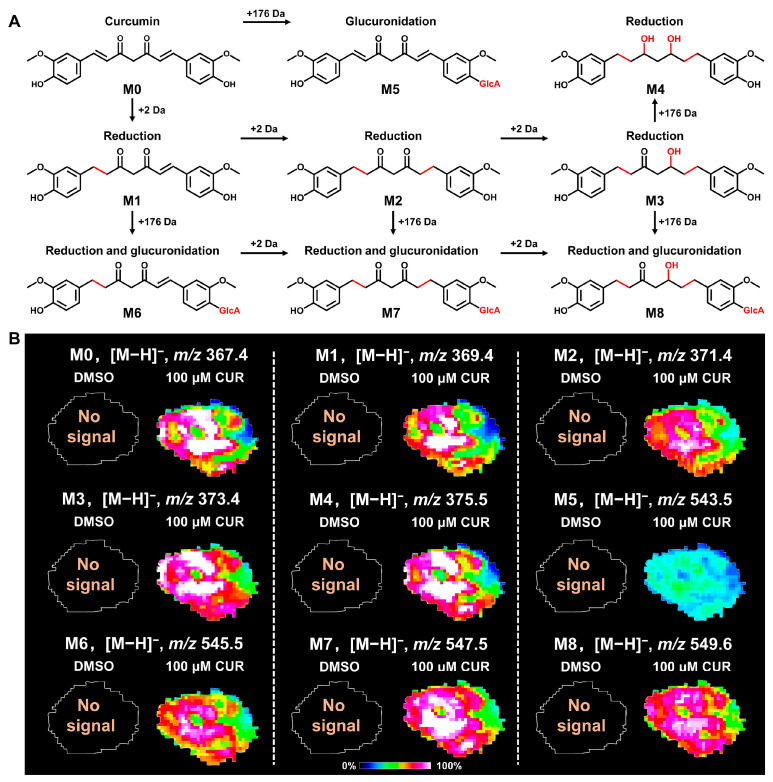
Spatial distribution of curcumin and its metabolites in tumor spheroids. (**A**) Metabolic pathway of curcumin in tumor spheroids. The red color indicates the parts that have undergone structural changes. (**B**) MALDI−MSI images of curcumin and its metabolites in spheroid sections. CUR: curcumin, M1: curcumin, M2: dihydrocurcumin, M3: tetrahydrocurcumin, M4: hexahydrocurcumin, M5: octahydrocurcumin, M6: curcumin glucuronide, M7: dihydrocurcumin glucuronide, M8: tetrahydrocurcumin glucuronide, M9: hexahydrocurcumin glucuronide.

To gain a better understanding of drug metabolism in solid tumors, it is essential to characterize the spatial distribution and changing patterns of drugs and their metabolites in 3D tumor spheroids. In this study, we utilized MALDI-MSI to map the spatial distribution characteristics of curcumin in tumor spheroids. In addition, we also detected and visualized the phase I and phase II metabolites of curcumin in the spheroid sections. Figure 3A illustrates the metabolic pathway of curcumin in tumor spheroids. We found that curcumin is metabolized intracellularly through dehydrogenation to dihydrocurcumin, tetrahydrocurcumin, hexahydrocurcumin, and octahydrocurcumin. These metabolites that underwent phase I metabolism can be further metabolized via glucuronidation to curcumin glucuronide, dihydrocurcumin glucuronide, tetrahydrocurcumin glucuronide, and hexahydrocurcumin glucuronide. Notably, there was no evidence of the sulfated metabolites of curcumin in spheroids. This is due to the fact that curcumin, once metabolized into curcumin sulfate, is not retained in cells but rather rapidly excreted [32]. Furthermore, we also found that curcumin could penetrate deeply into the interior of spheroids, with the highest content in the necrotic zone, followed by the quiescent zone and proliferative zone. These results provide a reliable deduction that curcumin can penetrate into the interior of solid breast cancer tumors and exert pharmacological properties.

### 3.4. Spatial Metabolomics Emphasizes Metabolic Alterations in 3D Tumor Spheroids

**Figure 4:**
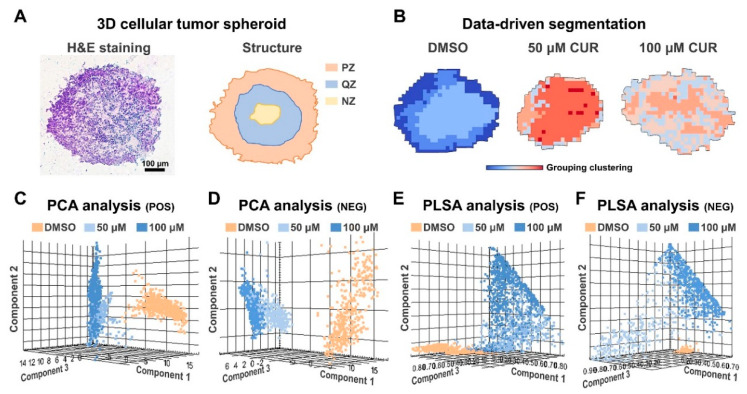
Curcumin−-induced metabolic alterations in 3D tumor spheroids. (**A**) H&E staining and structural diagram of 3D tumor spheroids. (**B**) Data-driven segmentation analysis. (**C**,**D**) PCA in positive− and negative−ion mode. (**E**,**F**) PLSA in positive− and negative−ion mode. PZ: proliferation zone, QZ: quiescent zone, NZ: necrotic zone, CUR: curcumin.

In order to further investigate the metabolic alterations of 3D tumor spheroids, we conducted a comprehensive spatial metabolomics analysis on spheroid sections. Initially, we utilized H&E staining to characterize the overall distribution of spheroids. As shown in Figure 4A, the H&E staining image of spheroids presented the zoning characteristics of the proliferation zone, quiescent zone, and necrotic zone, highlighting the spatial heterogeneity of spheroids. Subsequently, we extracted metabolite data utilizing MALDI-MSI and obtained data-driven segmentation images (Figure 4B). Metabolites with similar metabolic profiles were grouped together into similarly colored pixels. It can be seen that the colors of each group showed a gradient variation from inside to outside, which further emphasized the spatial distribution characteristics of the spheroids. Specifically, compared to the DMSO group, 50 μM CUR and 100 μM CUR groups exhibited varying shades of red, whereas the DMSO group showed variable shades of blue, demonstrating that curcumin plays a significant role in metabolic alterations on tumor spheroids. In addition, the extracted MALDI-MS spectra were subjected to in situ unsupervised principal component analysis (PCA) and probabilistic latent semantic analysis (PLSA) (Figure 4C–F). The results revealed a distinct clustering and grouping trend between the DMSO and curcumin groups in both positive- and negative-ion modes, thus providing clear evidence that spheroids underwent significant metabolic differences. The similar yet distinct clustering characteristics in the 50 μM CUR and 100 μM CUR groups with PCA and PLSA demonstrated that different degrees of metabolic changes occurred in two groups. Taken together, significant metabolic reprogramming occurred in 3D tumor spheroids after curcumin intervened, which warranted us to further investigate in order to elucidate the underlying mechanisms.

### 3.5. Curcumin Modulates Lipid Synthesis and Metabolism in 3D Breast Cancer Spheroids

**Figure 5:**
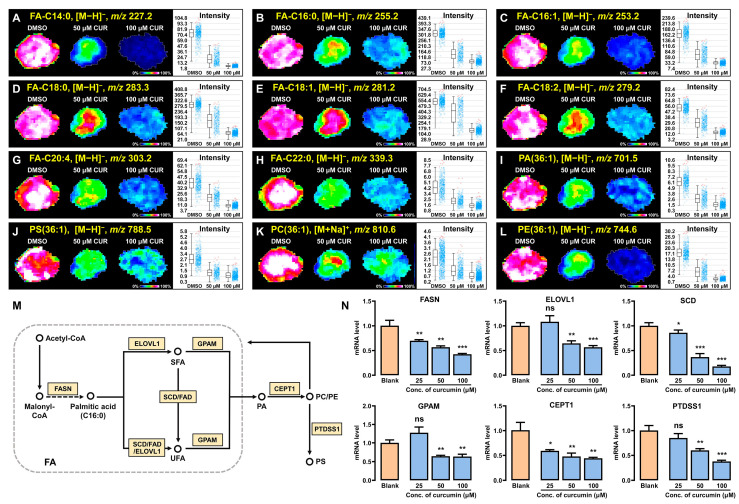
Curcumin−induced lipid metabolic reprogramming. (**A**–**L**) MSI images and relative contents of representative lipids. (**M**) Synthetic and metabolic pathways of lipids in 3D breast cancer cellular spheroids. (**N**) mRNA levels of key enzymes in lipid metabolism. Data are expressed as mean ± SD (*n* = 3). The yellow boxes in (**M**) represent the gene names code for key enzymes, and the circles represent metabolites. * *p* < 0.05, ** *p* < 0.01, *** *p* < 0.001, ns: not significantly different. SFAs: saturated fatty acids, UFAs: unsaturated fatty acids.

It is widely recognized that lipids are essential to the energy supply of cancer cells. Cancer cells exploited the oxidative metabolism of lipids to produce energy-rich phosphates, such as ATP, which provide vast amounts of energy necessary for proliferation and survival [33]. Here, our MALDI-MSI data revealed significant metabolic reconfigurations of fatty acids (FAs) and phospholipids such as phosphatidic acids (PAs), phosphatidylcholine (PC), phosphatidylethanolamine (PE), and phosphatidylserine (PS) in 3D tumor spheroids after curcumin induction. Furthermore, we characterized the spatial distribution characteristics of the representative metabolites in 3D tumor spheroids (Figure 5A–L). Interestingly, there was a significant downregulation in the relative contents of these lipids in spheroids after the curcumin intervention. Based on this phenomenon, we speculated that curcumin would be an effective inducer of lipid metabolic remodeling.

To further validate our hypothesis, we delved into the lipid synthesis pathways significantly altered in spheroids and determined the mRNA levels of key enzymes. As an important link in the process of lipid biosynthesis in organisms, a decrease in fatty acid synthesis can create conditions for inhibiting the development and proliferation of tumor cells. Specifically, fatty acid synthase (FAS) progressively converts Malonyl-CoA to palmitic acid (FA-C16:0) in a sequential reaction. Then, in the presence of the elongation of very-long-chain fatty acids protein 1 (ELOVL1), palmitic acid is prolonged to produce saturated fatty acids (SFAs) such as FA-C18:0, FA-C22:0, etc. Stearoyl-CoA Desaturase (SCD) or fatty acid desaturase (FAD), further catalyzes SFAs to FA-C16:1, FA-C18:1, FA-C18:2, FA-C20:4, and other unsaturated fatty acids (UFAs) (Figure 5M). Research shows that FASN is a crucial target for lipid metabolism in tumors [34,35]; inhibiting its activity can lessen the dependence. Notably, our quantitative real-time PCR results (Figure 5N) showed that curcumin effectively inhibited the gene expression of FASN, SCD, and ELOVL1. This demonstrated that curcumin exerted an inhibitory effect on fatty acid synthesis. In addition, phospholipids are involved in the regulation of key tumor signaling pathways such as cell proliferation, migration, invasion, and apoptosis, as well as play a critical role in inter-cellular signaling and the maintenance of cell membrane stability and function in cancer cells [36,37]. By evaluating the mRNA levels of these key enzymes related to phospholipid synthesis, we found a correlation between their expression levels and the abundance of lipid metabolites. Notably, their expression levels were significantly decreased following curcumin intervention (*p* < 0.05). In conclusion, curcumin can induce the metabolic reprogramming of lipid substances by modulating lipid synthesis and metabolism, thereby affecting proliferation, metastasis, and other processes in tumor spheroids.

### 3.6. Curcumin Regulates Polyamine Synthesis and Metabolism in 3D Tumor Spheroids

**Figure 6:**
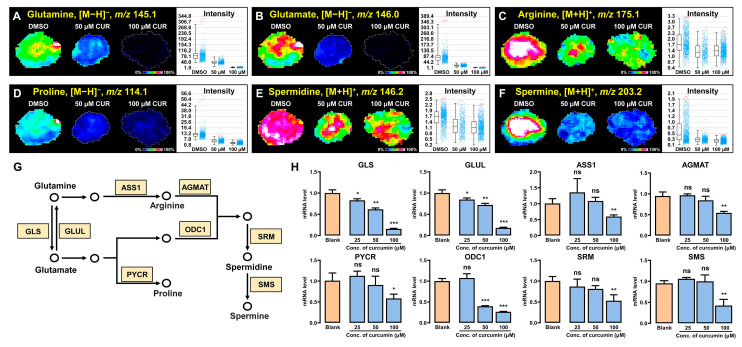
Curcumin regulates the anabolism of polyamines in 3D breast cancer cell spheroids. (**A**–**F**) MALDI−MSI images of glutamine, glutamate, arginine, proline, spermidine, and spermine in 3D breast cancer cell spheroids. (**G**) The polyamine synthesis and metabolism pathways that undergo major changes in cell spheroids. The yellow box represents the gene names that regulate key enzymes, while the circle represents metabolites. (**H**) mRNA levels of related enzymes in polyamine metabolism. * *p* < 0.05, ** *p* < 0.01, *** *p* < 0.001, ns: not significantly different.

Polyamine synthesis and regulation have important implications for the vital movement of cancer cells. Cell populations with an immunosuppressive phenotype, like triple-negative breast cancer cells, rely on elevated intracellular polyamine pools to sustain their growth and metabolism. Modulating the available polyamines levels can prevent tumor proliferation and reduce the likelihood of tumor immune escape, thus aiding anticancer treatment [38]. Our study found that the pathways implicated in polyamine synthesis were suppressed following curcumin intervention. Metabolic levels of certain precursors involved in polyamine synthesis, including glutamine, glutamate, arginine, and proline, exhibited a notable downregulation (Figure 6A–D). Glutamine and glutamate assume pivotal roles in the polyamine synthesis pathway, where deficiencies or impediments in their transport mechanisms may exert significant repercussions on polyamine synthesis and levels. Glutamine undergoes enzymatic conversion facilitated by glutaminase (GLS) and arginine succinate synthase 1 (ASS1) to yield glutamate and arginine, respectively. Moreover, glutamate can be catalyzed by pyrroline-5-carboxylate reductase (PYCR) to proline. These sequential processes provided a necessary material basis for polyamine synthesis (Figure 6G). The PCR results corresponded to the visualized images from the in situ analysis, demonstrating that curcumin inversely regulated the expression of GLS, GLUL, ASS1, and PYCR genes, thereby inhibiting key enzyme activities, resulting in the low expression of metabolites such as glutamine. Spermidine and spermine, pivotal metabolites in polyamine metabolism, exert a substantial influence on cellular proliferation and viability [39,40]. Arginine undergoes a series of reactions to produce spermine catalyzed by spermine synthesis (SRM), which in turn can react with proline to produce spermine by spermine synthesis (SMS). Figure 6E,F,H illustrate that curcumin not only downregulated the metabolic phenotypes of spermidine and spermine but also exerted a negative modulation on the gene expression levels of these key enzymes. Our results suggested that curcumin can intervene in the polyamine synthesis and metabolism pathway by regulating the activities of related enzymes, thus inhibiting the proliferation and metabolism of cancer cells and exerting antitumor properties.

## 4. Conclusions

In this study, we utilized a 3D tumor spheroids model and a mass spectrometry imaging technique to investigate the remodeling effect of curcumin on metabolism in MDA-MB-231 cells. Curcumin mainly inhibits the life course of cancer cells by interfering with their proliferation, migration, and other vital life activities. Using MALDI-MSI technology, we achieved high-resolution in situ visualization of metabolites in tumor spheroids after curcumin intervention, including lipids as well as polyamine synthesis and metabolism pathways. Our findings reveal the complex mechanism of curcumin in regulating the metabolic network of tumor spheroids, providing a critical theoretical basis for its prospective development as a potent antitumor therapeutic agent.

## Data Availability

The data presented in this study are available within the article and in the Supplementary Materials.

## Supplementary Materials

The following supporting information can be downloaded at: https://www.mdpi.com/article/10.3390/metabo14090482/s1, Figure S1: (A) Two-dimensional cell viability after intervention with tetrahydrocurcumin. (B) Spheroids growth area treated with different concentration of curcumin. Data are expressed as mean ± SD (*n* = 4). *** *p* < 0.001, ns: not significantly different; Table S1: Real-time PCR primer sequences.
