# Supplementary material for: Spatial Metabolomics Profiling Reveals Curcumin Induces Metabolic Reprogramming in Three-Dimensional Tumor Spheroids
Source: Metabolites. 2024 Sep 2;14(9):482. doi: 10.3390/metabo14090482 (PMC11433860; doi:10.3390/metabo14090482)
Supplement: Supplementary file 1 [file metabolites-14-00482-s001.zip › metabolites-3159576-supplementary.pdf]

Supplementary data

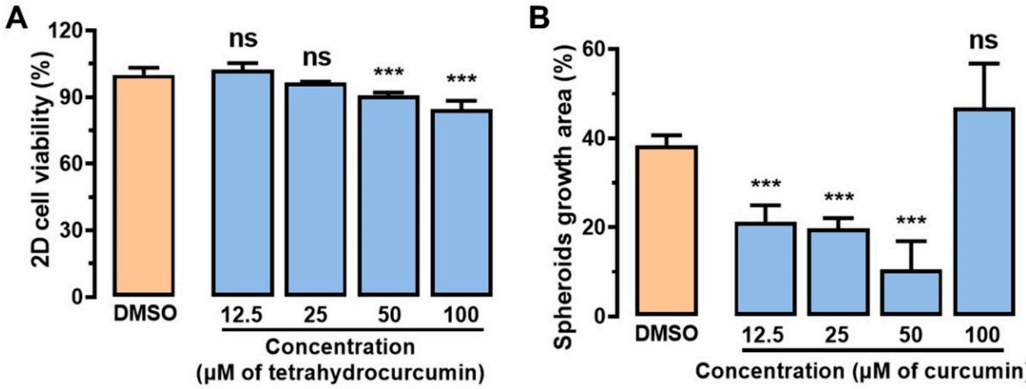

Figure S1. (A) 2D cell viability after intervention with tetrahydrocurcumin. (B) Spheroids growth area treated with different concentration of curcumin. Data are expressed as mean ± SD (n = 4). ***$P < 0.001$, ns: not significantly different.

Table S1. Real-time PCR primer sequences.

Gene	Forward	Reverse
β-actin	5'-CCAGACTCATTCAACCAGACA-3'	5'-GATGACTGAGTACCTGAACCG-3'
FASN	5'-CCTGGCTGCCTACTACATCG-3'	5'-CACATTTCAAAGGCCACGCA-3'
SCD	5'-TTCCCGACGTGGCTTTTTCT-3'	5'-AGCCAGGTTTGTAGTACCTCC-3'
ELOVL1	5'-CTTTGCCAGGCACCTTTTCC-3'	5'-TCTCTCGTCGGGGAACTCAG-3'
GPAM	5'-GAAGCTGGAGCTGCTAGGG-3'	5'-CCCAAATCATGTGCTGGGATG-3'
CEPT1	5'-TATTGTGCGCACTGGCAAAC-3'	5'-GCCAAAAAGGTGGTCCTCCA-3'
PTDSS1	5'-TCGCCTTTACCAGGGATGAC-3'	5'-CACAGCATACTCCATTGGGGA-3'
GLS	5'-GGGAATTCACTTTTGTCACGATCT-3'	5'-AAGGAATGCCTTTGATCACCAC-3'
GLUL	5'-TCGAGAGTGGGAGAAGAGCG-3'	5'-GTGGAAGGTGTTCTGGTCGC-3'
ASS1	5'-GCTTATAACCTGGGATGGGCA-3'	5'-TTGCTGGACATAGCGTCTGG-3'
AGMAT	5'-GTTGCAGACCTAGGCGATGT-3'	5'-TGTGTGATCTCCACCCAAGG-3'
PYCR1	5'-GGAGGTGGAAGAGGACCTGA-3'	5'-CATCTTGGCAGCCCCGTAG-3'
ODC1	5'-CTGGGCGCTCTGAGATTGTC-3'	5'-CCAGCTTCTCACAAAGGCAAC-3'
SRM	5'-TTGGACGGTGTCATCCAGTG-3'	5'-CCCCGATGATCAGCACCTTT-3'
SMS	5'-GCCAAAGCTGATGGTGAGAC-3'	5'-TATGGGTGGTAATCTGCCGT-3'
